# Targeting p21-activated kinase 1 for development of a novel anti-arrhythmic drug class

**DOI:** 10.1098/rstb.2022.0285

**Published:** 2023-06-19

**Authors:** Yu He, Alexander Grassam-Rowe, Ming Lei, James S. H. Bae

**Affiliations:** Department of Pharmacology, University of Oxford, Oxford OX1 3QT, UK

**Keywords:** p21-activated kinase 1, Ca^2+^ clock, membrane clock, protein phosphatase 2A, arrhythmia, FTY720

## Abstract

Evidence accumulated over the past decade suggests that p21-activated kinase 1 (PAK1) is a critical cardiac-protective signalling molecule. The present article provides an updated review of recent findings regarding the role of PAK1 in maintaining normal cardiac electrophysiological function through its regulation of membrane and Ca^2+^ clocks. We first overviewed the PAK1 activation mechanism. We then discussed recent updated results showing the action mechanisms of PAK1 signalling on Cav1.2/Cav1.3 (I_CaL_)-mediated Ca^2+^ entry, ryanodine receptor type 2-mediated sarcoplasmic reticulum (SR) Ca^2+^ release, transcriptional regulation of SR Ca^2+^-ATPase 2a, Na^+^/Ca^2+^ exchangers, and Ca^2+^/calmodulin-dependent protein kinase II. Finally, we proposed a new and exciting route for developing a PAK1-based therapeutic strategy for cardiac arrhythmias.

This article is part of the theme issue ‘The heartbeat: its molecular basis and physiological mechanisms’.

## 1. Introduction

Ventricular tachyarrhythmias (VTs) cause a significant mortality burden in worldwide and occur in both those with or without cardiac diseases, such as cardiac hypertrophy (CH) and heart failure (HF). Approximately 1 00 000 people a year die of VTs in the UK, of which about 50% of them are HF patients. Despite a large number of basic and clinical investigations, current pharmacotherapy is still inadequate, necessitating novel therapeutic approaches [1].

In this review, we discuss recent evidence for a critical role of p21-activated kinase 1 (PAK1) in maintaining normal cardiac electrophysiological function through its regulation of membrane excitability and intracellular Ca^2+^ homeostasis, in particular, as a critical regulator of membrane/Ca^2+^ clocks and as a novel potential therapeutic target for developing novel anti-arrhythmic drugs.

## 2. Two-clock theory for cardiac arrhythmogenesis

Two interacting clocks of cyclical changes in membrane potential and sarcoplasmic Ca^2+^ levels produce normal cardiac function. In a previous work, we proposed that at the physiological state, a membrane voltage clock (M-clock) modulates surface ion channels and electrophysiological changes, therefore, generates rhythmic spontaneous action potentials (APs) and conduction of excitation. Beyond the M-clock, a spontaneous Ca^2+^ release from the sarcoplasmic reticulum (SR) and Ca^2+^ reuptake occurring via SR Ca^2+^-ATPase 2a (SERCA2a) have been revealed to be the critical mechanism for sinus rhythm generation. These rhythmic alterations of sarcoplasmic Ca^2+^ concentrations give rise to the Ca^2+^ clock (C-clock), which dynamically interacts the membrane clock [2]. The M-clock feeds forwards onto the C-clock to regulate intracellular Ca^2+^ cycling. These cycles of intracellular store Ca^2+^ release and re-uptake are normally driven by the activation and recovery processes in the M-clock. Additionally, the C-clock also feeds back onto the M-clock through the Ca^2+^ modulating surface membrane molecules, causing the alterations of AP conduction, recovery, post-recovery stability, as well as exerting long-term effects on channel expressions [2]. The interactions of the C- and M-clocks to produce normal electrophysiological function are exemplified by the role of inward depolarizing Na^+^/Ca^2+^ exchange coupling Ca^2+^ cycling onto the sino-atrial node (SAN) automaticity [3]. The two-clock model also explains the autonomic parasympathetic and sympathetic cardiac regulation mechanisms through both coupled and independent modulations of the M- and C-clocks, respectively [2]. Thus, our two-clock model integrates subcellular physiology through to systems level as a heuristic model of cardiac function.

**Figure 1.**
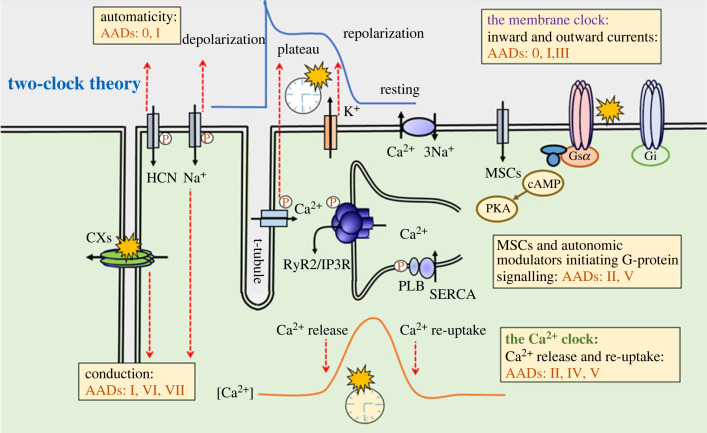
Cellular components involved in cardiomyocyte electrophysiological function and arrhythmic events and their grouping (AADs 0-VII) by pharmacological targets according to the updated Oxford classification scheme [4]. The two-clock theory is also presented in this schematic figure to illustrate the interaction between membrane (M) clock and Ca^2+^ (C-) clock for normal and abnormal cardiac activity [2]. SERCA, sarcoplasmic reticulum Ca^2+^-ATPase; RyR2, cardiac ryanodine receptor, type 2; PLB, phospholamban; PKA, protein kinase A; MSC, mechanically sensitive channel; Nav1.5, cardiac Na + channel protein; HCN, hyperpolarization-activated cyclic nucleotide-gated channel; G_s_, stimulatory G-protein; G_i_, inhibitory G-protein; Cx, connexin; cAMP, cyclic 3' 5-adenosine monophosphate.

Dysregulation of the two clocks gives rise to cardiac dysrhythmia. This two-clock theory model (figure 1) proposes that dysrhythmias can arise directly from abnormal M-clock function: either altered automaticity of the cardiac conduction system or from aberrant excitation or conduction in working cardiomyocytes. However, C-clock dysfunction could lead to potential pro-arrhythmic consequences as coupling to ion channel components in the M-clock. For example, increasing Ca^2+^ release through ryanodine receptor type 2 (RyR2) can produce rising cytosolic [Ca^2+^]. This dysregulated Ca^2+^ release could stimulate the propagating waves of spontaneous sarcoplasmic Ca^2+^ release, therefore, decoupling M- and C-clock activity. Moreover, this initial C-clock dysregulation may temporarily elevate electrogenic Na^+^/Ca^2+^ exchange current as the cytosolic [Ca^2+^] rises. This altered Na^+^/Ca^2+^ exchange current may then drive delayed after-depolarizations and disrupt ectopic excitation from M-clock [3]. Beyond the acute effects, C-clock dysfunction also feeds through to the M-clock through long-term transcriptional changes, such as downregulating longer-term Na^+^ channel expression [5]. Thus, the dyssynchronization of the C- and M-clocks gives rise to dysfunctional rhythmogenesis.

The two-clock theory provides a new heuristic foundation for development of anti-arrhythmic therapies. The earliest systematic approach to anti-arrhythmic therapies focused on the components of the C- and M-clocks primarily in isolation [4]. However, more recent attempts have provided a view more integrated with the two-clock theory [6]. The modernized classification retains the original organization in terms of isolated targets being discussed but expands the original classes I–IV from Vaughan Williams as: AP activation (class I), autonomic action (class II), AP recovery (class III), and Ca^2+^ current (class IV) [6]. The modernized classification expands on these classes taking recent updated findings into account; for example, late Na^+^ current targets were included in class I. Late Na^+^ current represents a recently proposed novel pro-arrhythmic mechanism in the failing human myocardium [7]. It also adds class 0, V, VI and VII agents acting on sino-atrial automaticity, mechanosensitive channels and connexin-regulated channels, as well as upstream modulatory targets [6]. Thus, through the systematic inclusion of molecular targets that compose the two-clock model, many more drug targets are brought into a coherent model of cardiac arrhythmogenesis.

## 3. p21-activated kinase 1 signalling in cardiac myocytes

The p21-activated kinases (PAKs) are a family of serine/threonine protein kinases. The PAKs were originally discovered as through their activation by downstream effectors of p21 proteins, small GTPases Cdc42 and Rac1 [8–10]. They are involved in critical roles of maintaining cytoskeleton dynamics, intracellular signalling and gene expressions [11–13].

**Figure 2.**
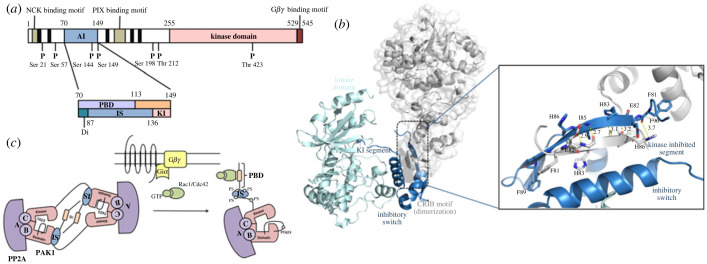
(*a*) Domain structure and autophosphorylation sites of the p21-activated kinase 1 (PAK1), containing 545 amino acids [14]. Proline-rich motifs represented by black bars. PIX interaction motif (182–203) and Nck binding motif (13–21) are presented in grey. The seven autophosphorylation sites are represented by P. KD, kinase domain (249–545); AID, auto-inhibitory domain (70–149). The detailed substructure of the autoregulatory region is shown in the expanded lower part of the diagram. PBD, p21 binding domain; IS, inhibitory switch domain; KI, kinase inhibitory. (*b*) Ribbon diagram of the inactive PAK1 dimer [13]. One PAK1 complex is in grey, the other, AID is in dark blue and KD is in light blue. Right diagram shows the detailed view of the dimer interaction. Yellow dotted lines show main chain hydrogen bonds between the two β strands of the dimerization segment. (*c*) Interaction between PAK1 and PP2A [14]. In the autoinhibited conformation, PAK1 is an asymmetric dimer and forms complexes with PP2A holoenzyme (A, conserved regulatory subunit; B, variable regulatory subunit; C, catalytic subunit). Binding of GTP-loaded Cdc42 (or Rac1) (green) with the PAK1 PBD dissociates the dimer and unfolds the IS domain, as well as induces the autophosphorylation of Thr423 (PT423). PAK1 autophosphorylation further triggers a conformational shift of PP2A, which appears to have a significant effect on PP2A localization.

Among the PAKs, PAK1 has been regarded to be the key enzyme in regulating the clocks for maintaining cytoplasmic homeostasis. PAK1 contains 545-amino acids, with a large NH_2_-terminal binding domain, an auto-inhibitory region (aa 70–149), and a COOH-terminal kinase domain (aa 255–529) (figure 2*a*) [15–17]. PAK1 is highly expressed in the blood vessels, brain and heart [18]. Specifically in the heart, ventricular cardiomyocytes exhibit significant PAK1 expression, delineating the nuclear membrane, intercalated disc, and Z-discs. Upon activation, PAK1 migrates to localized cytoplasm, thereby activating its downstream targets in maintaining cardiac homeostasis during contractions [19].

The auto-inhibitory domain of PAK1 is critical to the asymmetry dimerization formation of PAK1 (figure 2*b*). Structurally, inactive PAK1 was shown to be a homodimer with an antiparallel conformation, where the C-lobe of the kinase domain of a monomer interacts with the N-terminal inhibitory switch region of the other, thereby stabilizing in the inactive state [20]. However, recent small angle X-ray scattering study revealed that solution-phase structure of full-length PAK1 is monomeric both before and after activation, which is in contrast with previous findings [15]. Owing to these conflicting nature of structures, further structural studies of PAK1 is necessary to confirm its mode of activation [21]. PAK1 adopts its active conformation upon binding with its relevant GTPase, GTP bound form of Cdc42 and Rac1 [15]. These GTPases bind to PAK1 at the CRIB site within the regulatory domain of PAK1. Upon PAK1 activation, conserved residues on PAK1 are then autophosphorylated at multiple sites and its activation allows for interaction with the cellular targets [8,22].

Cardiomyocyte Ca^2+^-handling is tightly regulated by reversible phosphorylations, which is balanced by phosphatases and protein kinases. Protein phosphatase 2A (PP2A), and 1 (PP1) are two such major phosphatases, while protein kinase A (PKA) is a major protein kinase in cardiomyocytes. Enhanced adrenergic signalling results in higher expression of cAMP, thus cAMP-dependent PKA activation occurs. In cardiomyocytes, ion channels activation leads to PKA phosphorylation, consequently activating PP2A and PP1. These allow for a rapid signal termination of PKA, which is critical for regulating intracellular Ca^2+^ flux and contractile proteins, like phospholamban (PLB). The mechanism of PLB activation is through its dephosphorylation by PP2A and PP1, where PP1 has been suggested to be involved in 60–70% of PLB dephosphorylations [23–25]. Beyond regulating the C-clock reuptake, PP2A and PP1 also take part in regulating ryanodine receptors and Ca^2+^-sensitive sarcomere proteins such as MyBP-C and cTnI [14,26].

A close interaction between PAK1 and PP2A has suggested a possible novel cardiac signalling pathway (figure 2*c*). Sheenan *et al*. showed that PAK1 dissociates by Cdc42/Rac1, and its interaction with PP2A causes further conformational changes in PP2A, which can increase its activity towards substrates [27]. The phosphorylation processes of PKA during sympathetic stimulation can be balanced by this regulation of the PAK1–PP2A pathway in the cardiomyocytes. PAK1–PP2A acts as an important pathway in regulating contractility through modulating both the myofilaments and Ca^2+^ homeostasis [18,19,25]. Specifically, PAK1 and PP2A activation have been shown to improve cardiac contractile function during ischemia-reperfusion (I/R) injury via regulating the phosphorylation levels of cTnT and MLC2 [28–30]. The PAK1–PP2A signalling pathway also modulates RyR, dihydropyridine receptors and PLB in control of SERCA activity and thereby regulates intracellular Ca^2+^ [31,32].

## 4. The role of p21-activated kinase 1 in regulating the Ca^2+^ clock

Recent studies have revealed the regulation of Ca^2+^ homeostasis under physiological and adrenergic stress conditions by PAK1 signalling pathways [33–35]. Overexpression of CA-PAK1 has been shown to change the Ca^2+^ transient decay constants, reducing the isoprenaline (ISO)-stimulated rise in L-type Ca^2+^ channel (I_CaL_) activity. Abnormal Ca^2+^ homeostasis such as elevated diastolic [Ca^2+^]_i_ and dysregulated SR Ca^2+^, especially under β-adrenergic stress conditions, were observed in cardiomyocyte-specific PAK1 knock-out (*PAK1^cko^*). These results demonstrate that the regulation of PAK1 activity in cardiomyocytes plays a critical role in maintaining Ca^2+^ homeostasis.

### (a) Potential role of p21-activated kinase 1 in regulating ryanodine receptor type 2 function

Further evidence suggests that PAK1 could have a critical role in maintaining normal RyR2 function via PP2A signalling. It is suggested that the phosphorylation of RyR2 is highly regulated by a macromolecular signalling complex that contains PKA, Ca^2+^/calmodulin-dependent protein kinase II (CaMKII) and the phosphatases (PP1 and PP2A). In addition, RyR2 hyperphosphorylation by PKA or by CaMKII are the major causes of Ca^2+^-dependent arrhythmia such as catecholaminergic polymorphic ventricular tachycardia (CPVT) and VTs associated with HF [36,37]. Such effect of phosphorylation however is more complex since the inability to phosphorylate RyR2 at S2808 also increases diastolic SR Ca^2+^ leaks [38,39]. It is hypothesized that the PP2A catalytic subunit could form complexes with the L-type Ca^2+^ channels and RyR2, and thereby reverses the phosphorylation done by PKA, CaMKII or protein kinase C (PKC) [40–43]. Interestingly, although the single-channel effects of PP1 have been demonstrated, the effects of PP2A on the gating of RyR2 channels have not yet been established [44–46]. The PP2A catalytic subunit has been proposed to bind to RyR2 directly through a leucine zipper motif. The regulatory subunit PR130 may also contribute to the association of the PP2A catalytic subunit to the RyR2 complex [47]. Studies have shown that an overexpression of microRNA miR-1 selectively decreases expression of the PP2A regulatory subunit, B56alpha, which results in an enhancement of cardiac excitation–contraction (E-C) coupling through upregulating the phosphorylation of the RyR2 and L-type Ca^2+^ channels [43].

Our recent study further indicates that RyR2 channels from *PAK1^cko^* hearts exhibit increased probability of opening. This has been evidenced by reverse transcription-quantitative polymerase chain reaction (RT-qPCR) and Western blot studies which showed pCamkII*δ* and RyR2 are highly phosphorylated at baseline in the atria of *PAK1^cko^* mice, while Slc8a2 and Slc8a3 expression are augmented. This demonstrates that the deficiency of PAK1 promotes atrial arrhythmogenesis under adrenergic stress, probably through post-translational and transcriptional modifications of key molecules that are critical to Ca^2+^ homeostasis [35].

The regulation of SR function by PAK1 was demonstrated through the transection of adenovirus expressing constitutively active PAK1 (Ad-CaPAK1) into cardiomyocytes. In Ad-CaPAK1 myocytes, the overexpression of Ad-CaPAK1 reversed the effect of ISO-induced SR Ca^2+^ release. A significantly decreased amplitude in [Ca^2+^]_i_ transient amplitude, a reduced rate of [Ca^2+^]_i_ decay and an increased SR Ca^2+^ content were observed after an ISO treatment [34]. Without the ISO treatment, spontaneous SR Ca^2+^ release sparks were dramatically downregulated in amplitude, and the Ca^2+^ overload was found to be reduced as well during the spontaneous Ca^2+^ contractions in Ad-CaPAK1 myocytes. Overall, these results show that the Ad-CaPAK1 expression plays an important role in sensitizing myofilaments and in stabilizing Ca^2+^ transients.

### (b) Potential role of p21-activated kinase 1 in regulating Ca^2+^ reuptake through transcriptional regulation of SERCA2a expression

Ca^2+^ flows back into the SR via SERCA2a, but this movement can be blocked by dephosphorylated phospholamban. Phosphorylated phospholamban by PKA at the Ser16 site subsequently attenuates its inhibition of SERCA2a and thus improves the reuptake of Ca^2+^ into the SR [18]. PAK1 has been shown to modulate SERCA2a expression via the transcription factor, serum response factor (SRF) [48]. Specifically, activation of PAK1 could lead to SRF nuclear translocation, and further studies in neonatal rat cardiomyocytes (NRCMs) infected with Ad-CaPAK1 have demonstrated SRF as the intermediate responsible for PAK1-regulated transcriptional modulation of SERCA2a. This has been supported by the evidence that *PAK1^cko^* hearts showed an associated impairment of SERCA2a function and downregulation of SERCA2a messenger RNA (mRNA) and protein expression. PAK1 was revealed to be the necessary component in maintaining ventricular Ca^2+^ homeostasis and electrophysiological stability, functioning as a novel regulator of cardiac SERCA2a through transcriptional mechanisms [48]. Previous studies have indicated that bradykinin can promote PAK1 autophosphorylation and dephosphorylate phospholamban via PP2A, leading to inhibition of Ca^2+^ reuptake by the SR and thus, slowing Ca^2+^ transients [28]. In addition, expression of phosphorylated phospholamban protein has been measured to change in Ad-CaPAK1 transfected myocytes, suggesting PAK1's reversibility in PKA-induced phospholamban phosphorylation and hindering Ca^2+^ uptake through SR [34].

Isolated mouse ventricular myocytes (VMs) with PAK1 deficiency (PAK1*^cko^*) were used to explore PAK1's underlying mechanism on arrhythmic activity during simulated ischemia. PAK1*^cko^* VMs showed an exaggerated boost in [Ca^2+^]_i_, leading to spontaneous Ca^2+^ released. However, the Ca^2+^ overload in PAK^cko^ VMs could be attenuated by the administration of a reverse mode blocker (KB-R7943) of the Na^+^/Ca^2+^ exchanger (NCX) or a Rac1 inhibitor (NSC23766). Suppressed PAK1 activity in PAK1*^cko^* VMs or VMs treated with the PAK1 inhibitor (IPA3) were shown to promote greater cellular reactive oxygen species (ROS) production than those of the control group. Voltage clamp recordings also demonstrated higher NCX activity in PAK*^cko^* VMs which indicated greater ROS production through NOX channel activity. Overall, these studies suggest that PAK1 is an important negative regulator of NOX2 and thereby attenuates ROS production [49].

## 5. The role of p21-activated kinase 1 in membrane clock

### (a) p21-activated kinase 1 regulation of Ca^2+^ channels

In SAN pacemaker cells, the major surface membrane molecules and the ion currents are generated from various ion channels, including hyperpolarization-activated cyclic nucleotide-gated, NCX, I_CaL_, T-type Ca^2+^ channels (I_CaT_) and delayed rectifier K^+^ channels (I_K_) [4]. Activation of these ion channels target downstream molecules such as PKA, PP2A and PP1. PP2A is revealed to regulate the L-type Ca^2+^ current (I_CaL_) through a reduction in I_CaL_ and E-C coupling gain, as indicated in voltage-clamped myocytes dialyzed with PP2A [50]. More evidence has shown that PKA agonists could regulate the activity of L-type Ca^2+^ channels via PKA-induced phosphorylation [51]. A selective PP2A inhibitor, fostriecin, was shown to normalize the t-tubule I_CaL_ density in ISO-treated ventricular myocytes, indicating that PP2A plays an important role in suppressing the activity of L-type Ca^2+^ channels during ISO stimulation [52]. In addition, the application of okadaic acid, a PP2A inhibitor, in Ad-CaPAK1 transfected SAN cells showed an increase in I_CaL_ induced by ISO, suggesting that PAK1 can modulate L-type Ca^2+^ channels via interacting with PP2A [31]. Moreover, S1P was suggested to modulate intracellular Ca^2+^ handling though L-type Ca^2+^ channels via the PAK1–PP2A signalling pathway. In rat ventricular myocytes, S1P was shown to decrease frequency of spontaneous Ca^2+^ waves and I_CaL_ amplitude, triggered by ISO. This was thought to be the effect of S1P in activating PAK1 and causing co-localization of PAK1 and PP2A, consequently attenuating the beta-adrenergic signalling pathway [32]. This was further supported by DeSantiago *et al*. where they showed that PAK1 is involved in critical roles of regulating E-C coupling in ventricular myocytes and in structural maintenance of the T-tubular system—which is critical for hypertrophic remodelling [53].

### (b) p21-activated kinase 1 regulation of small conductance Ca^2+^-activated K^+^ channels

A recent study by Yang *et al*. showed a change in expression of ventricular Ca^2+^ -activated K^+^ (SK2) channel and its function in hypertrophied ventricular myocytes, specifically in NRCMs and in intact murine hearts [54]. Angiotensin II (Ang II) was applied to both the control group (PAK1*^f/f^*) and the PAK1 knocked-out group (shRNA-PAK1 interference or PAK1*^cko^*). Interestingly, Ang II treatment had minimal effect on the SK2 currents in PAK1*^cko^*, compared to that of the PAK1^f/f^ isolated cardiomyocytes. Both the histology and echocardiography showed greater hypertrophic measurement in PAK1*^cko^* than that of PAK1*^f/f^* cardiomyocytes. The reduced cardiac contractility was observed to be parallel with the increased expression of SK2 protein, increased hypertrophic indices, and reduced cardiac contractility. The increased SK2 protein expression in ventricular myocyte was changed more in PAK1*^cko^* in comparison to that of the PAK1*^f/f^*.

In NRCMs, Ang II stimulation confirmed such increases in apamin-sensitive SK patch clamp currents and cell hypertrophy, while real-time PCR and Western blot results replicated the changes in both the mRNA and protein expression levels of SK2. Furthermore, functional studies measuring myocyte hypertrophy was shown to be induced by shRNA-PAK1 interference, yet this was attenuated by the application of the PAK1 agonist, FTY720. Interestingly, elevated CaMKII and CREB phosphorylation were found to accompany these effects. These changes were reversed by FTY720 as well as KN93, a CaMKII inhibitor [54]. Further luciferase assays confirmed that the CREB specifically binds to the KCNN2 promoter sequence, modulating its expression levels. These results indicate that the elevated SK2 expression in Ang II-induced hypertrophic model was potentially through the CaMKII/CREB signalling pathway, in convergence with the PAK1 pathway, and consequently increased the KCNN2 promoter activity [54].

The expressed SK2 can provide a novel mechanism linking the levels of cytosolic Ca^2+^ through its binding with calmodulin (CaM). This Ca^2+−^ CaM binding then interacts with SK2 C-terminal domains and activates SK channels. Intracellular Ca^2+^ levels thus have a similarly CaMKII-dependent action on the expressed SK2. This process would downregulate ADP leading to a decreased extracellular Ca^2+^ entry and SR Ca^2+^ release to compensate for the abnormal Ca^2+^ level. CaMKII-dependent signalling was also reported to be important in cardiomyocyte hypertrophy. Its nucleocytoplasmic transfer was shown regulating L-type Ca^2+^ channel, mediating Ca^2+^ influx in cultured hippocampal neurons [54]. However, more evidence is needed to demonstrate the inhibitory mechanism of CaMKII on PAK1 and to better understand its function in maintaining intracellular Ca^2+^ homeostasis under cardiac hypertrophic conditions.

## 6. p21-activated kinase 1 as a therapeutic target for designing novel anti-arrhythmic drugs

### (a) Abnormal automaticity is the major mechanism for the initiation and maintenance of triggered ventricular tachyarrhythmias

Abnormal automaticity is responsible for both the initiation and maintenance of a major type of VTs, referred as ‘triggered ventricular tachyarrhythmia', that results from aberrant Ca^2+^ handling from the SR in ventricular myocytes. Over the past two decades, significant progress has been made in understanding the mechanisms responsible for Ca^2+^ handling with both normal (AP-induced, systolic) and abnormal (spontaneous, diastolic, Ca^2+^ overload-related) SR Ca^2+^ release. The latter has been linked to both inherited arrhythmia syndromes as a result of genetic defects in RyR2 (e.g. CPVT) and acquired forms of heart disease (e.g. CH and HF) or adrenergic stress [5,36,55,56]. Although many molecules are involved in abnormal diastolic SR Ca^2+^ release and related VTs, aberrant opening of RyR2 during diastole is an ‘essential' component. Therefore, factors that increase the likelihood that RyR2 channels open during diastole (when they should be closed to allow for replenishment of SR Ca^2+^ stores) predispose the heart to dysrhythmia [5,36,55,56]. In patients with structural heart disease, such as CH and HF, acquired alterations in RyR2 function occur primarily due to post-translational modification of the channels. There is extensive evidence that chronic changes in phosphorylation of RyR2 in CH and HF are associated with an increased SR Ca^2+^ leak [57]. RyR2 can be phosphorylated at multiple sites PKA, PKC, and CaMKII [44,58]. Many of those sites are contained within or near a single flexible loop [59,60]. There is controversy over the relative importance of the various phosphorylation sites but antibodies recognizing the phosphorylated state of S2808, S2814 and S2030 are useful tools that that can provide an indication of the degree to which RyR2 is phosphorylated by PKA and CaMKII [41,61–63]. Many studies have linked alteration in RyR2 phosphorylation with inappropriate diastolic SR Ca^2+^ release leading to delayed-after-depolarizations that in turn trigger Ca^2+^-dependent VTs [57]. In addition, a universal characteristic of hypertrophied and failing myocardium is depressed SERCA2a function owing to decreased expression or increased inhibition by the protein PLB, or both [64]. This gives rise to reduced SR Ca^2+^ load, and elevated diastolic [Ca^2+^]_i_.

Factors that prevent abnormal diastolic SR Ca^2+^ release should attenuate Ca^2+^-dependent VTs [65]. Current anti-arrhythmic drugs that typically focus on plasma membrane ion channels have shown limited clinical success, and in some cases have even been described as being pro-arrhythmic. Targeting pathological abnormal SR Ca^2+^ release via cardiac RyR2 provides an additional avenue for anti-arrhythmic therapy. This may be a promising target to potentially increase survival in patients with acquired heart diseases.

### (b) p21-activated kinase 1 regulation in arrhythmia

**Figure 3.**
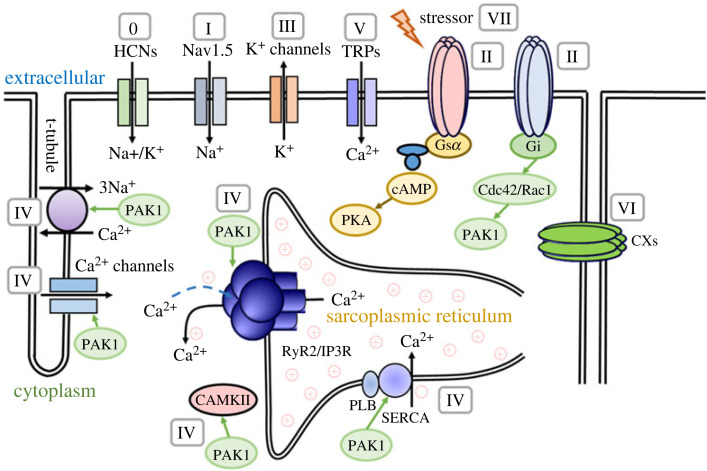
The potential action mechanisms for activation of PAK1 signalling in antiarrhythmias according to the updated Oxford classification scheme [4]. Class IV actions: through its phosphatase-mediated effects on Cav1.2/Cav1.3 (I_CaL_) mediated Ca^2+^ entry, RyR2-mediated SR Ca^2+^ release, transcriptional regulation of SERCA2a, Na^+^/Ca^2+^ exchangers (class IVd) and CaMKII. Class VII actions: it has longer-term modifications of arrhythmic substrate including electrophysiological and/or structural remodelling for primary or secondary prevention.

Given the critical role of PAK1 in regulating in both M-clock and C-clock in cardiac myocytes, we anticipate that PAK1 can be a feasible target for developing a novel class of anti-arrhythmic drugs. Figure 3 illustrates the potential action mechanisms for activation of PAK1 signalling in antiarrhythmias. Class IV actions: through its phosphatase-mediated effects on Cav1.2/Cav1.3 (I_CaL_) mediated Ca^2+^ entry, RyR2-mediated SR Ca^2+^ release, transcriptional regulation of SERCA2a, NCX (class IVd) and CaMKII. Class VII actions: it has longer-term modifications of arrhythmic substrate including electrophysiological and/or structural remodelling for primary or secondary prevention, reducing susceptibility to or progression of established atrial and ventricular arrhythmias, particularly in the presence of cardiac failure and hypertension.

Previous studies have provided strong evidence for supporting such hypothesis. For example, overexpression of constitutively active PAK1 altered Ca^2+^ transient decay constant (*τ*_Ca_) [46], and promoted anti-adrenergic signalling through attenuation of ISO-induced increases in I_CaL_ and phospholamban phosphorylation both probably through PP2A activation. We also showed that a bioactive peptide derived from the PAK1 auto-inhibitory region increases PAK1 activity and counteracts Ang II-induced pathological hypertrophy and ventricular arrhythmias [36].

Moreover, previous research has also indicated that FTY720 (Fingolimod), an immuno-modulatory agent, activates PAK1 and prevents I/R injury-induced arrhythmias via activated PAK1/Akt signalling pathways in studies of rat heart subjected to I/R. In addition, FTY720 can induce nitric oxide (NO) production via the PI3K/Akt/eNOS signalling pathway, which is also reported to be important for cardiac protection [66]. FTY720 was also found to prevent Ang II-induced arrhythmic Ca^2+^ overload by attenuating NCX activity in a NOX2-dependent manner in atrial myocytes from a canine model of ventricular tachypacing-induced atrial fibrillation [67].

Recent studies have also demonstrated that FTY720 reduces myofilament Ca^2+^ responsiveness and improves diastolic dysfunction and partially reverses atrial remodelling in a mouse model treated with a hypertrophic cardiomyopathy-linked mutation in tropomyosin [66]. Ryba *et al*. have demonstrated that the modulation of S1PR through FTY720 led to reduced myofilament-Ca^2+^-responsiveness and improved diastolic function in hypertrophic cardiomyopathy might be owing to decreased oxidative modification of myofilament proteins via downregulation of NOX2 [68]. Furthermore, FTY720 was shown to reverse Ang II induced increase of SK2 expression and cell hypertrophy in NRCMs [52]. These cardioprotective effects of FTY720 against cardiac arrhythmias, I/R injury as well as the prevention and reversal of hypertrophy, have demonstrated that PAK1 is a very promising pharmacological target of heart diseases.

## 7. Conclusion and perspective

There are a large amount of studies for the PAK1 enzyme function in regulating neurodevelopment, neuroplasticity as well as the metastasis and cell motility of cancer cells [69–71]. For example, loss of PAK1 has been indicated to be associated with the progression of neurodegenerative disorders, particularly Alzheimer's disease [72]. However, explorations for the effects of PAK1 in the heart were only initiated two decades ago.

Increasing remarkable studies have demonstrated the critical role of PAK1 in the heart from its regulation in the membrane clock and Ca^2+^ homoeostasis to protect against cardiac arrhythmias. These findings promote future development of PAK1 activators as a potential novel therapeutic strategy for both prophylactic and therapeutic measures involving cardiac arrhythmia. Since PAK1 is involved in the pathogenic and oncogenic effects, as well as PAK inhibitors, which may be developed and deployed in cancer therapy, anti-viral infection, including COVID-19, and other diseases, critical questions are raised to discuss how to balance the cardiac-protective role of PAK1 activation and the anti-tumour effect of PAK1 inhibition in drug discovery research [73–76]. Interestingly, FTY720, components of the PAK1 signalling pathway, have been demonstrated a proven efficacy in multiple *in vitro* and *in vivo* cancer models, suggesting a potential therapeutic role in cancer patients [77]. Therefore, continued investigation of the different regulation pathways of PAK1 in various disease models would provide an opportunity to offer input and guidance on new directions for PAK1 in pathogenic and drug discovery research.

## Data Availability

This article has no additional data.
